# National and sub-national trends of salt intake in Iranians from 2000 to 2016: a systematic analysis

**DOI:** 10.1186/s13690-022-00871-w

**Published:** 2022-04-13

**Authors:** Ali Gholami, Ali Ghanbari, Shahabeddin Rezaei, Hamid Reza Baradaran, Shahab Khatibzadeh, Mahboubeh Parsaeian, Mitra Hariri, Negar Zamaninour, Ali Sheidaei, Morteza Abdollahi, Parvin Mirmiran, Majid Ghayour-Mobarhan, Afshin Ostovar, Noushin Mohammadifard, Alireza Khosravi, Seyedeh Mahdieh Namayandeh, Farshad Farzadfar

**Affiliations:** 1 Noncommunicable Diseases Research Center, Neyshabur University of Medical Sciences, Neyshabur, Iran; 2 Department of Epidemiology, School of Public Health, Iran University of Medical Sciences, Tehran, Iran; 3 Non-Communicable Diseases Research Center, Endocrinology and Metabolism Population Sciences Institute, Tehran University of Medical Sciences, Tehran, Iran; 4 Ageing Clinical and Experimental Research Team, Institute of Applied Health Sciences, School of Medicine, Medical Sciences and Nutrition, University of Aberdeen, Aberdeen, UK; 5 The Heller School for Social Policy and Management, Brandeis University, Waltham, MA USA; 6 Department of Epidemiology and Biostatistics, School of Public Health, Tehran University of Medical Sciences, Tehran, Iran; 7 Minimally Invasive Surgery Research Center, Iran University of Medical Sciences (IUMS), Tehran, Iran; 8 Social Determinants of Health Research Center, Shahid Beheshti University of Medical Sciences, Tehran, Iran; 9 Nutrition and Endocrine Research Center, Research Institute for Endocrine Sciences, Shahid Beheshti University of Medical Sciences, Tehran, Iran; 10 Metabolic Syndrome Research Center, School of Medicine, Mashhad University of Medical Sciences, Mashhad, Iran; 11 Osteoporosis Research Center, Endocrinology and Metabolism Clinical Sciences Institute, Tehran University of Medical Sciences, Tehran, Iran; 12 Isfahan Cardiovascular Research Center, Cardiovascular Research Institute, Isfahan University of Medical Sciences, Isfahan, Iran; 13 Hypertension Research Center, Cardiovascular Research Institute, Isfahan University of Medical Sciences, Isfahan, Iran; 14 Research Center of Prevention and Epidemiology of Non-Communicable Diseases, Shahid Sadoughi University of Medical Sciences, Yazd, Iran; 15 Endocrinology and Metabolism Research Center, Endocrinology and Metabolism Clinical Sciences Institute, Tehran University of Medical Sciences, Tehran, Iran

**Keywords:** Salt intake, Urinary sodium, Dietary, Iran

## Abstract

**Background:**

One fifth of the global burden of cardiovascular diseases (CVDs) in 2017 was attributable to excessive salt intake. As a member of the World Health Organization (WHO), Iran has committed itself to a 30% reduction in salt intake by 2025. Evidence on the amount and trend of salt intake among the Iranian population at national and sub-national levels is scarce. This study aimed to estimate the Iranian population’s salt intake during 2000–2016 at the national and sub-national levels, by sex and age groups.

**Methods:**

Data on national and sub-national mean salt intake was obtained through systematically searching the literature and contacting the research studies’ principal investigators. Data collected through various methods were harmonized using the cross-walk method. Bayesian hierarchical and spatio-temporal-age regression models and simulation analysis were used to estimate the mean salt intake and its uncertainty interval across sex, age, year, and province.

**Results:**

National age-sex standardized mean salt intake decreased from 10·53 g/day (95% uncertainty interval [UI]: 10·2 to 10·9) in 2000 to 9·41 (9·2 to 10·6) in 2016 (percent change: − 9·8% [− 21·1–3·1]). The age-standardized mean salt intake in women had decreased from 9·8 g/day (95% UI: 9·0–10·6) in 2000 to 9·1 g/day (8·6–9·7) in 2016 (percent change: − 6·6% [− 19·0–7·9]). The same measure in men was 11·1 g/day in 2000 (95% UI: 10·3–11·8) and 9·7 g/day (9·1–10·2) in 2016 (percent change: − 12·7% [− 23·0 – -0·9]).

Age-sex standardized mean salt intake at the sub-national level in 2016 varied from 8·0 (95% UI: 7·0–9·0) to 10·5 (10·0–11·1). The difference between the provinces with the highest and the lowest levels of salt intake in 2016 was 31·3%.

**Conclusion:**

Salt intake decreased in Iran from 2000 to 2016, while persistently exceeding the recommended values. This declining trend was more pronounced between 2010 and 2016, which might be attributed to Iran’s compliance to WHO’s Action Plan for reducing NCDs.

**Supplementary Information:**

The online version contains supplementary material available at 10.1186/s13690-022-00871-w.

## Key messages

➢ Mean salt intake decreased in Iran from 2000 to 2016.

➢ National and sub-national age-standardized mean salt intake decreased in Iran from 2000 to 2016 among women and men.

➢ The largest decrease in salt intake occurred in the youngest age group (25–34-year-olds).

➢ The percentage of Iranians who consumed salt more than double the WHO recommended value dropped from 2000 to 2016.

## Background

Excess salt intake is among the leading risk factors for non-communicable diseases (NCDs), particularly in cardiovascular diseases (CVDs) and gastric cancer [1]. In addition to raising blood pressure (BP), excess salt intake may exert its detrimental effects by inducing the release of pro-inflammatory cytokines, development of obesity and insulin resistance [2, 3]. According to the Global Burden of Disease study in 2017, approximately 2·7 million deaths from CVDs and 327,000 deaths from gastric cancer were attributable to excess salt intake [1].

Owing to the fact that there is a dose-response relationship between salt intake and BP [4], reduction of salt intake is a cost-effective approach toward reducing BP and thereby reducing CVDs risk [5, 6]. Based on available evidence, reduction of salt intake down to the level recommended by the World Health Organization (WHO) and other relevant organizations (5–6 g/day), causes significant reduction in BP [4, 7]. Accordingly, reduction of salt intake has become one of the voluntary global targets of WHO Action Plan for the Prevention and Control of NCDs 2013–2020 [8].

As a member of WHO, Iran has committed itself to reduce salt intake by 30% at the national level by 2025. To design appropriate policies and evaluate the effect of current salt reduction interventions, the trends of salt intake at the national and sub-national levels need to be estimated; however, no data regarding the trend of salt intake in Iran is available. Therefore, to address the knowledge gap, this study was performed to estimate the Iranian population’s salt intake across a 17-year period at the national and sub-national levels and within sex and age subgroups.

## Methods

This study contains four steps:

- Systematic literature search for retrieving articles published on salt or sodium intake
- Gathering data from all national and sub-national (provincial, district and community) epidemiologic studies possessing data on salt or sodium intake by contacting the studies’ principal investigators
- Data preparation
- Statistical analysis

### Systematic literature search for retrieving published articles on salt or sodium intake

A systematic literature search was performed using international electronic databases (PubMed, Web of Science and Scopus), and national electronic databases (Scientific Information Database, IranMedex and IRANDOC) for studies which were conducted from January 1st 2000 to December 31st 2016. Moreover, Medical Subject Headings (MeSH) were used in PubMed for each keyword. The database syntax is presented in Table 1 of the Appendix, on page 9.

Two phases of screening were performed by two autonomous reviewers to assess the eligibility of the retrieved studies. In the first step, the articles’ titles and abstracts were assessed, and in the next step, their full-texts were assessed; those that did not meet the eligibility criteria were discarded. Any disagreements were discussed and resolved upon consensus and consulting an expert. The eligibility criteria are presented in the Eligibility Criteria section of the Appendix, on page 2.

The National Institute of Health (NIH) quality assessment forms were used for quality assessment of the articles [9]. The forms assess potential flaws in study methods or implementation, including sources of bias, confounding, study power, the strength of causality, and other factors.

In the next step, the required data including general characteristics of the studies and salt intake data were extracted from the included studies (Data Extraction section of the Appendix, on page 3).

### Gathering data from national and sub-national studies

In addition to retrieving data through literature review, we obtained data from studies that had measured salt/sodium intake in Iran by contacting the studies’ principal investigators. We also used Household Income and Expenditure Surveys (HIES) (2004–2016) data in this study (National and Sub-national Studies Data section of the Appendix, on page 3).

#### Data preparation

The methods of salt intake measurement varied across different studies, including 24-h urine sodium, spot urine sodium, 3-day food record, food frequency questionnaire (FFQ), food recall, and HIES. We categorized methods of data collection into three groups, including: 1- Urinary sodium and three-day food record along with weighting, 2- Food Frequency Questionnaire (FFQ) and food recall, and 3- HIES. The first category was considered as the gold standard method. In studies that reported sodium intake, we multiplied the sodium value by 2·54 to convert it to salt. To calculate salt intake from HIES, we converted the food items of HIES data to individual intake using a method described by Imhoff-Kunsch [10]. Then, we extracted sodium values for each food item (more than 200 food items) using the modified Iranian Nutritionist IV software, then, total daily sodium intake for each individual was calculated.

We cross-walked between obtained values of salt/sodium in different areas (urban, rural) using linear regression models to achieve representative data for the provinces. Also, a hierarchical linear model (nested for province, sex and age groups) was used to cross-walk the values of salt/sodium in different methods of data collection (the abovementioned three categories), which provides weighted salt/sodium intake regardless of the type of data collection. Details of conversion (cross-walk) regressions and further information on the methods of data collection are presented in the Data Preparation section of the Appendix, on page 6–7. The inverse variance of salt intake in different methods was used to minimize the variance of the weighted average of salt values obtained in different methods. We then provided a frame that included years of study (2000–2016), provinces studied (31 provinces in Iran), sex (male-female) and age groups (seven age groups: 25–34, 35–44, 45–54, 55–64, 65–74, 75–84 and ≥ 85) and merged the prepared data into this frame (18,414 rows).

#### Statistical analysis

After data preparation, we found that certain provinces lacked salt data or representative data at provincial level during some years of the study. In some surveys, we only had data for one sex or a few age groups. Two models were used in this study, which are described in more detail in the Appendix. First, we used the weighted Bayesian multilevel model with three levels of nested structures (age groups, within sex groups, within provinces). We included some important covariates including urbanization, years of schooling, wealth index, and temperature in the fixed effects structure of the constructed model. We also included intercept and two quadratic time trend parameters in the random effects structure*.* Second, we applied the age-spatio-temporal model [11] using residuals of posterior samples in the first model to address age-spatio-temporal dependencies. Finally, we estimated the median salt intake and its 95% uncertainty interval (UI) from the 2·5th and 97·5th percentiles based on the second model’s results, across sex, age, year, and province. Iran’s national population in 2016 –tabulated by sex and age- was used to remove the age effect (age-standardization).

In order to assess the validity of the model in estimating the mean salt intake, we performed a sensitivity analysis. In this regard, we randomly masked 10% of our data points and then, we repeated all the models for the remaining 90% of data. We repeated this process five times. Then, we computed the percentage of the overlap between confidence intervals of the masked data and uncertainty intervals of estimates of the model repeated for the remaining 90% of data. All statistical analyses were conducted in STATA and R software.

## Results

**Fig. 1:**
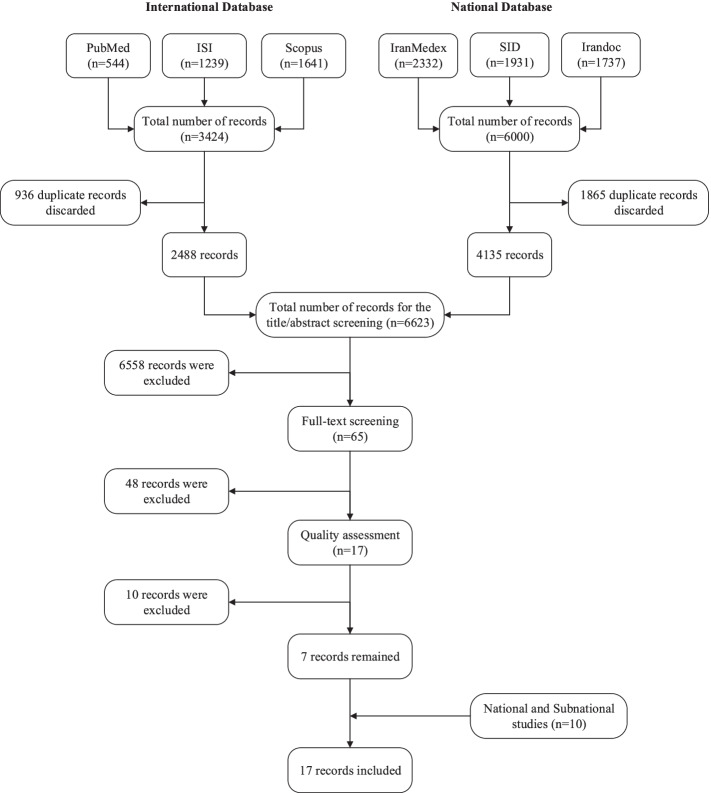
Flowchart of search for published articles and national and sub-national studies on salt or sodium

Overall, we gathered data from seven published articles [12–18] and 10 national and sub-national studies (Fig. 1 & Supplementary Table 2); seven reported 24-h urinary sodium data, one reported spot urinary sodium data and nine reported dietary salt/sodium data, with more than one and half million participants. In total, 5975 data points were generated after collapsing data based on year, province, sex and age groups. All the provinces had at least three data sources during certain years. The numbers of data points for each province and year are presented in Supplementary Fig. 1.

### Distribution of salt intake by year, province, sex, and age

**Fig. 2:**
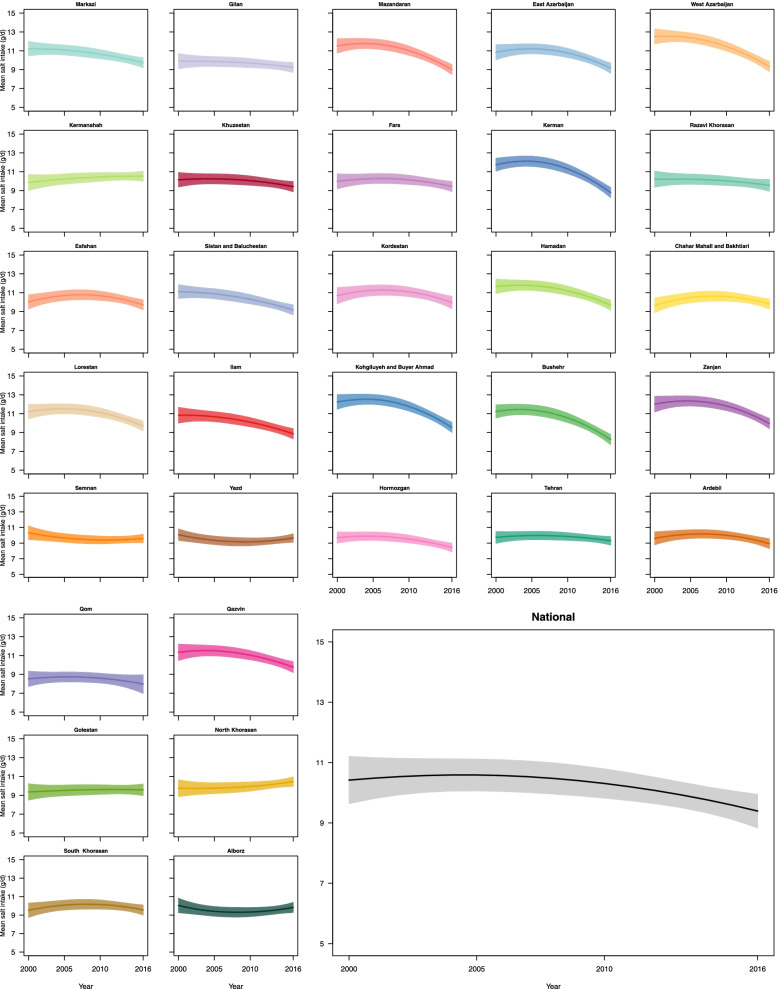
Trends of age-sex-standardized mean salt intake by province from 2000 to 2016 in Iran

Nationally, age-sex-standardized mean salt intake decreased from 10·53 g/day (95% UI: 10·2–10·9) in 2000 to 9·41 g/day (9·2–10·6) in 2016 (Fig. 2). Accordingly, the percent change in age-sex-standardized mean salt intake from 2000 to 2016 was − 9·8% (− 21·1–3·1). The percentage of people who consumed salt more than double the WHO recommendation were 52, 56, 52, and 25% in 2000, 2005, 2010, and 2016, respectively.

The age-standardized mean salt intake among women decreased from 9·8 g/day (95% UI: 9·0–10·6) in 2000 to 9·1 g/day (8·6–9·7) in 2016 (Supplementary Fig. 2). Similarly, in men, the age-standardized mean salt intake fell from 11·1 g/day in 2000 (95% UI: 10·3–11·8) to 9·7 g/day (9·1–10·2) in 2016 (Supplementary Fig. 3). Accordingly, the percent change in age-standardized mean salt intake from 2000 to 2016 was − 6·6% (− 19·0–7·9) in women and − 12·7% (− 23·0 – − 0·9) in men.

**Fig. 3:**
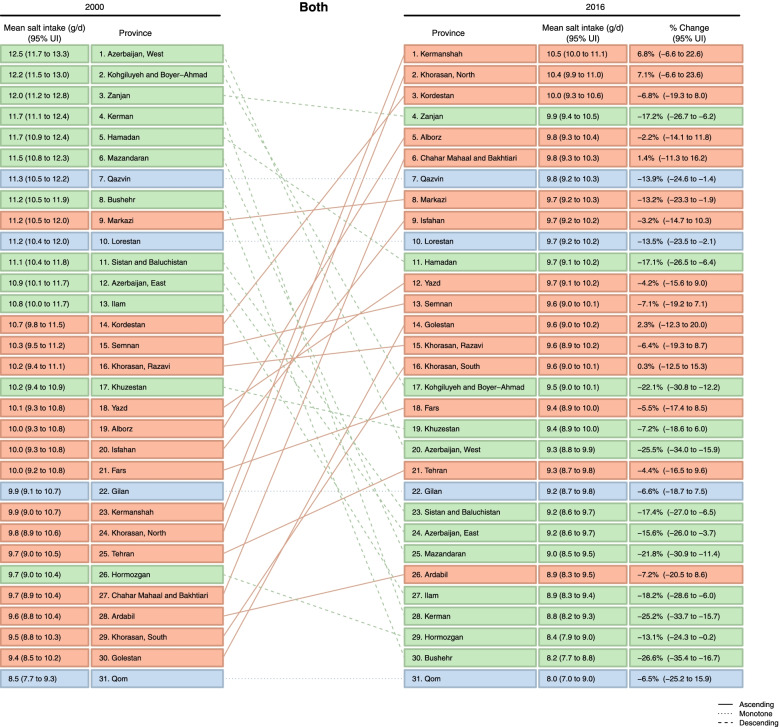
Ranking of age-sex-standardized mean salt intake by province in 2000 and 2016 in Iran

The variation of age-sex-standardized mean salt intake between provinces changed from 8·5 (95% UI: 7·7–9·3) to 12·5 (11·7–13·3) in 2000, and from 8·0 (7·0–9·0) to 10·5 (10·0–11·1) in 2016 (Figs. 2 and 3). The difference between the provinces with the highest and the lowest levels of salt intake was 47·1% in 2000, which dropped to 31·3% in 2016. The percent change in age-sex-standardized mean salt intake from 2000 to 2016 ranged from 0·3% to − 26·6%. The highest percent change (− 26·6%: 95% UI: − 35·4 – − 16·7) was observed in a southern Iranian province (Bushehr), in which the mean salt intake in 2000 was 11·2 g/day (10·5–11·9), and the lowest percent change (0·3%: − 12·5–15·3) was observed in a province northeast of Iran (South Khorasan), in which the mean salt intake in 2000 was 9·5 g/day (8·8–10·3) (Fig. 3).

**Fig. 4:**
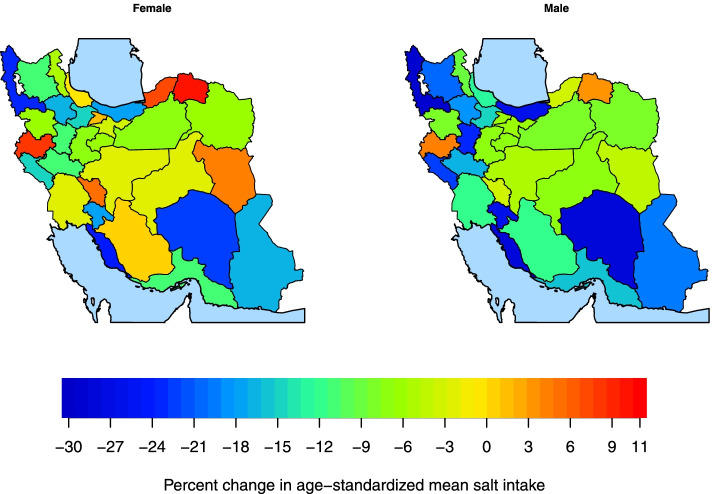
Percent change in age-standardized mean salt intake by sex and province from 2000 to 2016 in Iran

Sub-national age-standardized mean salt intake decreased in women and men over the period of the study (Supplementary Figs. 2 & 3). The percent change in age-standardized mean salt intake at sub-national levels from 2000 to 2016 ranged from 0·1% to − 23·9% in women and from − 2·4% to − 28·2% in men. The highest percent change in women (− 23·9%: 95% UI -33·3 – − 13·5) was observed in Bushehr (a southern province), in which the mean salt intake in 2000 was 10·5 g/day (9·9–11·2), and the lowest percent change (0·1%: − 13·6–16·2) was observed in Gilan (a northern province) in which the mean salt intake in 2000 was 9·0 g/day (8·2–9·8) (Fig. 4 & Supplementary Fig. 4). The highest percent change in men (− 28·2%: 95% UI -36·0 – − 19·3) was observed in West Azerbaijan, in which the mean salt intake in 2000 was 13·3 g/day (12·5–14·1), and the lowest percent change (− 2·4%: − 14·1–10·9) was observed in a Central province (Chaharmahal and Bakhtiari), in which the mean salt intake in 2000 was 10·1 g/day (9·4–10·9) (Fig. 4 & Supplementary Fig. 5).

**Fig. 5:**
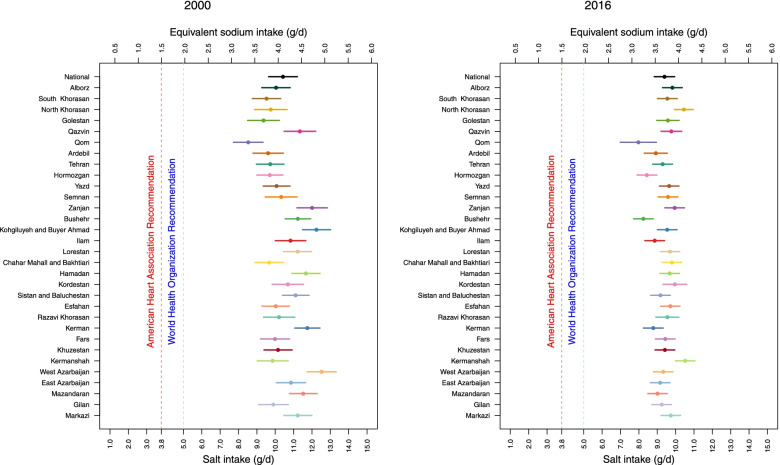
Age-sex-standardized means and 95% uncertainty intervals of salt intake in 2000 and 2016 in Iran

**Fig. 6:**
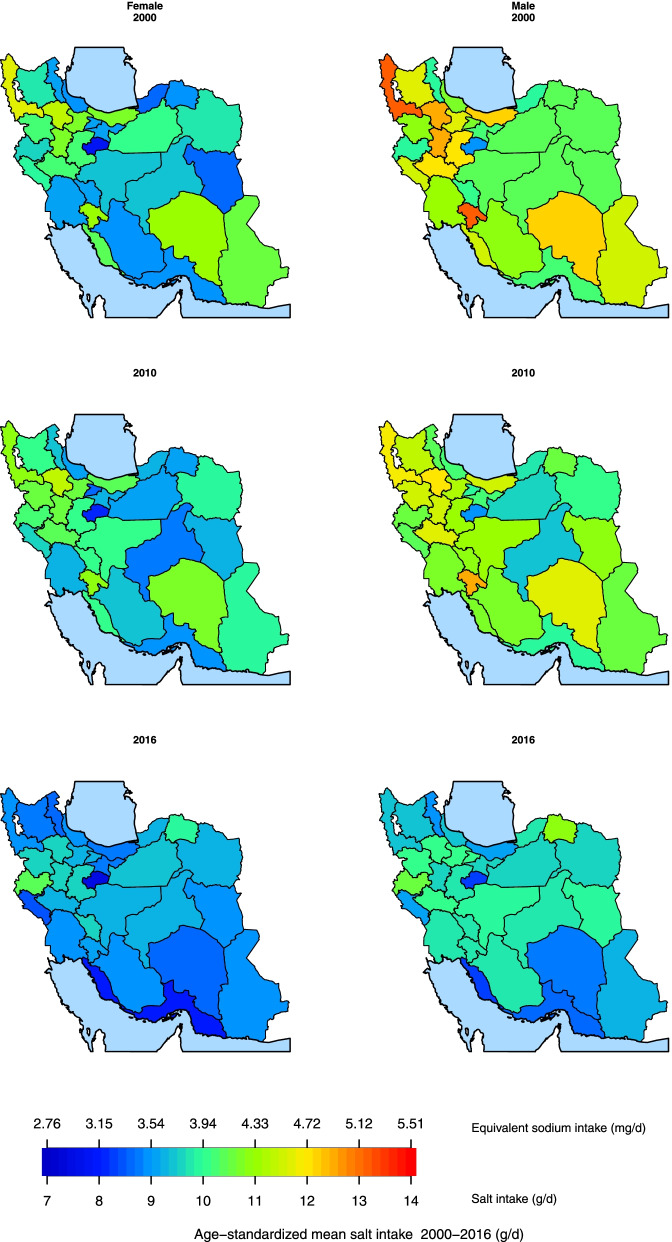
Age-standardized mean salt intake by sex and province in 2000, 2010 and 2016 in Iran

As presented in Fig. 5, the age-sex-standardized mean salt intake in 64·5% of the provinces was more than double the WHO recommendation in 2000. This value decreased to 6·5% in 2016. Age-standardized prevalence of very high salt intake (more than double the WHO recommendation) decreased sub-nationally from 41·9% to 29·2% in women and from 96·8% to 29·0% in men from 2000 to 2016, respectively (Fig. 6 & Supplementary Fig. 6), nevertheless, all provinces still had salt intake levels above the WHO recommended level.

**Fig. 7:**
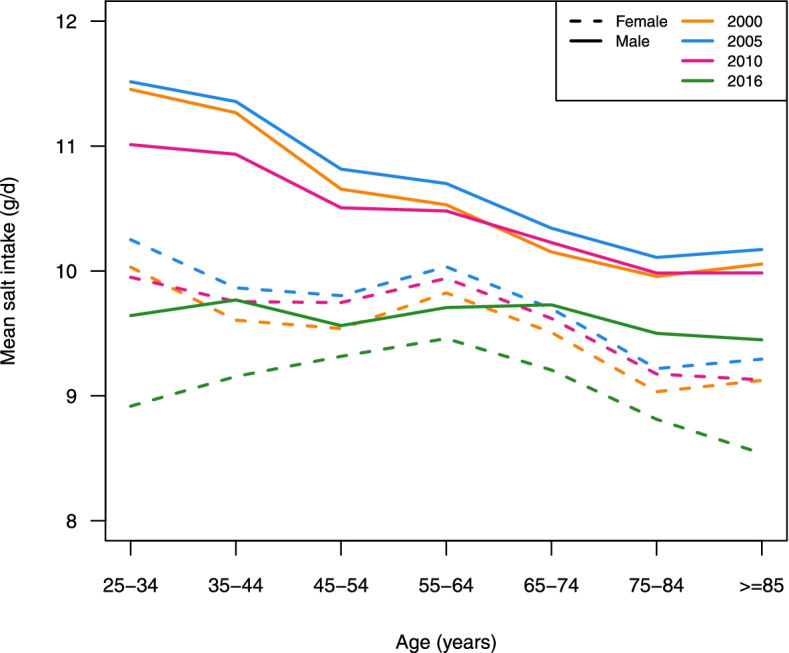
Age-standardized mean salt intake by sex and age groups in 2000, 2005, 2010 and 2016 in Iran

Mean salt intake decreased from 2000 to 2016 in each age group and in both sexes. The largest decrease in salt intake, which occurred in the youngest age group (25–34-year-olds), was 1·1 g/day for women and 1·8 g/day for men (Fig. 7).

Based on the sensitivity analysis, 86·5% of the observed and predicted data points had overlaps. Also, the Root Mean Square Error (RMSE) for the total data was 0·608 and for 90% of the data it was 0·630.

### Discussion

The present study provides, for the first time, the trend of salt intake at the national and sub-national levels in Iran. Nationally, after a slight increase in the early years of the study, the age-sex-standardized mean salt intake showed a declining trend (flatted inverted U shape) in Iran, yet it was approximately two times higher than the WHO recommendation for adults. This pattern was observed in both men and women.

The mean salt intake fell by 9·8% in Iran from 2000 to 2016. One of the main reasons behind the declining trends of salt intake might be related to governmental policies in recent years. The significance of excess salt intake –as one of the main risk factors for NCD development- has been taken into consideration in Iran’s National Action Plan for Prevention and Control of NCDs [19]. Salt reduction by 30% is one of the latter Plan’s targets by 2025 [19]. To reach this goal, several policies have been executed in recent years. Traffic light labeling is one of these policies. All food companies have been mandated to report the amount of salt/sodium, energy, sugar and total fat/trans-fatty acid in their products by using the traffic light colors indicating low (green), medium (yellow) and high (red) since 2016 [20]. Another policy was the reformulation of foods, particularly bread, that is one of the biggest contributors to salt intake in Iran [21]. In recent years, the amount of salt in breads was reduced by 50% in Iran [21, 22]. Last but not least, another policy was raising awareness regarding the detrimental effects of excess salt intake through mass media and public campaigns [21].

To date, national trends of salt/sodium intake have been investigated in a limited number of countries, including the United States, China, Japan, Finland, England and South Korea. Furthermore, most studies have reported the trends of salt intake up until 2010, before the WHO Action Plan for Prevention of NCDs. In the US, the mean sodium intake did not change significantly between 2001 and 2010. According to the findings of the National Health and Nutrition Examination Survey (NHANES), the mean salt intake among adults (aged 19–50-years-old) was 9·7 g/day in both NHANES 2001–2002 and NHANES 2009–2010 [23]. Similarly, the mean sodium intake had not changed significantly between 1998 and 2009 in South Korea. Based on the Korean NHANES, the mean salt intake was 11·7 g/day in 1998 and 11·9 g/day in 2009 [24].

In Finland, between 1979 and 2002, the mean salt intake decreased from 12·9 g/day to less than 9·9 g/day in men and from 10·5 g/day to less than 7·6 g/day in women [25]. In China, the mean salt intake decreased significantly from 1997 to 2011. According to findings from the China Health and Nutrition Surveys, the mean salt intake was 12·9 g/day, 12·2 g/day, 10·1 g/day, 9·3 g/day, 8·9 g/day and 8·4 g/day in 1997, 2000, 2004, 2006, 2009 and 2011, respectively [26]. A similar pattern was observed in Japan, wherein the mean salt intake had dropped between 1953 and 2014 [27]. Similarly, in England, the mean salt intake, measured by 24-h urinary sodium excretion, decreased significantly from 2003 to 2011. According to the findings of the Health Survey for England, the mean salt intake was 9·5 g/day in 2003, 9 g/day in 2005/2006, 8·64 g/day in 2008 and 8·1 g/day in 2011 [28]. The declining trends in these countries demonstrate their interventions’ success.

The WHO, according to available evidence on salt reduction policies and interventions, designed a SHAKE package to enable countries to achieve a reduction in salt intake [29]. The SHAKE package, which stands for *S*urveillance, *H*arness industry, *A*dopt standards for labelling and marketing, *K*nowledge and *E*nvironment, assists countries with the development, implementation and monitoring of salt reduction policies and interventions which have proved to be effective in reducing population salt intake [29]. In spite of Iran’s success in reducing salt intake in recent years, there is still a need for more effective policies and interventions involving the participation of other sectors, and in particular the food industry, to reach the 30% reduction in salt consumption targeted by the WHO. Pursuing the policies and interventions mentioned in the SHAKE package that have not been executed in the country might prove effective in the further lowering of salt intake by the population. Some of these interventions are as follows. 1) Periodically repeating the measurement of salt intake at national and sub-national levels as performed in STEPs 2016 [30]; 2) Measuring the salt content of food; 3) Setting target levels for the amount of salt in foods; 4) Reformulation of salty foods; 5) Adopting marketing regulations for foods containing high amounts of salt; 6) Continue to raise public awareness on the health risks of high salt consumption; 7) Educating people about the ways they can reduce their salt consumption; 8) Implementing multicomponent salt reduction strategies in community settings.

Furthermore, the findings of this study demonstrate that the mean salt intake was greater in men than in women. This finding is consistent with the pattern observed globally and in other countries [27, 31–36]. The reason behind the higher salt intake in men may be attributed to their higher overall caloric intake compared to women in Iran [37, 38]. However, reduction in salt intake was observed in all age groups during the study period in both sexes, which demonstrates that everyone, regardless of their age, benefits from salt reduction policies.

Previously, the mean sodium intake in Iran was estimated in 1990 and 2010 by Powles et al. [31] They reported that the mean sodium intake had increased from 3·85 g/day (equivalent salt intake: 9·8 g/day) in 1990 to 3·95 g/day (equivalent salt intake: 10·06 g/day) in 2010. The current estimation is more accurate than the value that was reported by Powles et al. in 2010 [31]. One of the main reasons is that, Powles et al. [31] included exclusively three studies that were about four provinces of Iran with the total sample size of 2239 participants, among which, two studies had measured dietary salt intake and one had measured salt intake via urine. These studies, which were included in Powles et al’ s study [31], had been performed in 1998, 2000 and 2001. However, in the current study, we included data from all provinces and from both rural and urban regions, for all the age groups, between 2000 and 2016 (Supplementary Fig. 1).

The strength of this study is that it is the first to produce comprehensive estimates of the Iranian population’s salt intake during the years 2000 to 2016 at the national and sub-national levels and within sex and age subgroups. We used data from the majority of earlier large-scaled studies and published articles. Most studies that have estimated salt intake have used a single data source for salt intake measurement. Moreover, we have searched several international and national databases. Another strength of this study is its investigation period. Here, we measured the trends of salt intake up to 2016. The majority of studies have measured trends of salt intake by 2010, before the WHO Action Plan. Using different statistical models (Bayesian hierarchical model, Spatio-temporal-age regression model and simulation analysis) to estimate the exact values of salt intake and their uncertainty intervals is another strength of this study.

One of the limitations of the current study is that primary data on some provinces and years were lacking, which has been addressed by the statistical models applied.

## Conclusion

In conclusion, Iranians’ salt consumption is more than double the WHO recommended value, though salt intake decreased in both men and women in Iran from 2000 to 2016. This declining trend occurred particularly between 2010 and 2016, which implies that Iran has been following the WHO Action Plan for reducing NCDs.

## Supplementary Information

**Additional file 1.**

**Additional file 2.** 

**Additional file 3.**

**Additional file 4.** 

**Additional file 5.**

**Additional file 6.** 

**Additional file 7.** 

## Data Availability

The source of data belongs to the Non-Communicable Diseases Research Center of Tehran, which is basically available to all researchers and academic institutes on a formal request based on transparency regulations.

## Abbreviations

CVD: Cardiovascular Diseases
NCD: Non-Communicable Diseases
BP: Blood Pressure
NIH: The National Institute of Health
FFQ: Food Frequency Questionnaire
HIE: Household Income Expenditure
RMSE: Root Mean Square Error
NHANES: National Health and Nutrition Examination Survey
WHO: World Health Organization
