# Supplementary material for: National and sub-national trends of salt intake in Iranians from 2000 to 2016: a systematic analysis
Source: Arch Public Health. 2022 Apr 13;80:120. doi: 10.1186/s13690-022-00871-w (PMC9006553; doi:10.1186/s13690-022-00871-w)
Supplement: Supplementary file 1 — Additional file 1. [file 13690_2022_871_MOESM1_ESM.docx]

**National and Sub-national Trends of Salt Intake in Iranians from 2000 to 2016: a systematic analysis**

Supplementary appendix

| **Table of contents** | |
| --- | --- |
| Overview | 2 |
| Data Sources | 2 |
| 1.Systematic Literature Search | 2 |
| - Eligibility Criteria | 2 |
| - Quality Assessment | 3 |
| - Data Extraction | 3 |
| 2. Data of Large-Scaled National and Sub-national Studies | 3 |
| Data Preparation | 4 |
| Statistical Model | 6 |
| Age Standardization of results | 7 |
| Model validity | 7 |
| References | 8 |
| Supplementary Table 1: Search Syntax | 9 |
| Supplementary Table 2: National and Sub-national surveys from 2000 and 2016 among Iranian population | 10 |
| Supplementary figures legend | 11 |

***Overview***

This study was conducted to estimate the trend of salt intake at the national and sub-national levels by sex and seven age groups (25-34, 35-44, 45-54, 55-64, 65-74, 75-84 and ≥85) during the period of 2000 to 2016. In the first step, a systematic literature search was performed for retrieving the articles reported mean salt or sodium intake. The retrieved articles were screened for eligibility of inclusion. The included studies were undergone quality assessment, and the required data were extracted from them. Besides finding the related studies through literature search, the data of the large-scale national and sub-national studies in Iran was obtained through communication with principal investigators of the studies. In the next step, the data of the salt intake of the included studies that measured through various methods were harmonized using cross-walk method. For estimation of the value of salt intake in some points that there were no data, Bayesian hierarchical model and Spatial-temporal-age regression model were used. Simulation analysis was used to estimate the uncertainty interval of each predicted salt intake point.

***Data Sources***

***1. Systematic Literature Search***

We searched the scientific literature databases for articles on salt or sodium intake until December 2016. The details of search syntax of the international databases are presented in Supplementary Table-1 of the appendix.

- ***Eligibility Criteria***

We included the nationally or sub-nationally representative studies providing data on mean salt or sodium intake. We only included studies that had random sampling, study population aged ≥25 years, performed between 1 January 2000 to 31 December 2016, and mentioned the province of study, area of study (rural or urban), year of study, sex, age and number of population. There was no language limitation, and we included the articles that were written in English and Persian. Studies were excluded if using non-random samples; conducted among specific subpopulations (e.g., specific employees, pregnant women, racial or ethnic minorities, immigrants, individuals with specific diseases, or volunteers); providing inadequate information on any of the inclusion criteria.

- ***Data Extraction***

The following data were extracted from each included paper:

1) Articles Unique ID (DOI or PMID), 2) First Author Name, 3) Publication Year, 4) Corresponding Author Name, 5) Corresponding Author Email, 6) Study Name, 7) Study Source, 8) Study Design, 9) Study Setting, 10) Province, 11) Study Population, 12) Sampling Unit, 13) Sampling Method, 14) Sampling Weight, 15) Representativeness, 16) Sample Size, 17) Response Rate, 18) Sex, 19) Age Range, 20) Age Mean/Median, 21) Age Standard Deviation, 22) Type of Data, 23) Date of Data Collection, 24) Data Collection Method, 25) Type and Value of Central Tendency for Salt/Sodium Intake, 26) Type and Value of Dispersion Measure for Salt/Sodium Intake and 27) Type of Bias.

***2. National and Sub-national Studies Data***

The large-Scaled studies that measured salt/sodium intake in Iran and included in this study are as follows: through contacting the principal investigator of the studies.

1. The WHO STEPwise approach to Surveillance (STEPS)
2. Comprehensive Project on Household Food Pattern and Nutritional Status
3. The Tehran Lipid and Glucose Study (TLGS)
4. Isfahan Healthy Heart Program (IHHP)
5. Isfahan Salt Study
6. Persian Gulf Healthy Heart Study (PGHHS)
7. MASHAD Study
8. Yazd Healthy Heart Program (YHHP)
9. Urban Health Equity assessment and Response Tool (Urban Heart-phase1)

In addition, we used Household income and expenditure surveys (HIEs) (2004-2015) in this study.

Details of the all included studies are summarized in Table-2 of the appendix.

***Data Preparation***

After data extraction of the studies retrieved through literature search, the outlier value of salt or sodium intake was identified through the following formula and omitted from the study.

$$x_{i}<Q_{1}-1.5IQR or x_{i}>Q_{3}+1.5IQR$$

Some of the studies merely investigated the mean salt or sodium intake at the rural or urban region. Therefore, the value of salt or sodium intake in those studies was not representative of the province. To make data representative of the province, multiple linear regression model was used. The variables “Year of Study”, “Age”, “Sex”, “Years of Schooling”, “Wealth Index” and “Temperature” were used as covariates in the mixed linear regression.

The methods of salt intake measurement of the included studies were varied, including 24-h urine sodium, spot urine sodium, 3-day food record, food frequency questionnaire (FFQ), food recall, and household income expenditure (HIEs). The data of the spot urine sodium was converted to 24-hour sodium through Tanaka equation ([1](#_ENREF_1)). To convert sodium to salt, we multiplied sodium by 2.54. Furthermore, since 90% of the sodium excreted through urine and the remainder is excreted via sweat, intestinal fluids, and saliva, we added extra 10% to the value of 24-hour salt intake ([2](#_ENREF_2)). The food items of HIEs data were converted to individual intake using Imhoff-Kunsch method ([3](#_ENREF_3)).

For homogenizing data of the different salt intake methods, we used the cross-walk method. The data of the 24-h urine sodium, spot urine sodium and 3-day food record methods considered as a standard method. Although, the gold standard for salt intake measurement is 24-h urine sodium ([4](#_ENREF_4)); however, due to paucity of data of urine sodium and after experts consultation we merged the data of mentioned methods and considered as a gold method (Group-1). After consultation with experts, the data of FFQ and food recall were merged and considered as Group-2. The data of the HIEs were considered as Group-3.

***Statistical Model***

Despite comprehensive literature search, and obtaining data of the large-scale national and sub-national studies, there were no data in some provinces in some years of study. The data points of the study in all the provinces of Iran and across the study period is demonstrated in Supplementary Figure-1 of the appendix. In two stages, salt intake was estimated. First, Weighted Bayesian Multilevel Model with three level nested structures was used. Age groups nested within sex groups, nested within provinces. The temporal trend of the data was of a quadratic nature. Intercept and two quadratic time trend parameters were included in the random effects structure and other covariate including urbanization, years of schooling, wealth index, a­­­nd temperature in the fixed effects structure. Four parallel chains were constructed, each with 2000 samples, and the first half of each chain was used as a warm-up and discarded.

The mixed model for the balanced three-way nested is given by

$$y_{ijkt}=\alpha+ \beta_{1}\times yos+\beta_{2}\times WI+\beta_{3}\times urbanization+\beta_{4}\times tempreture+$$

$$\omega_{i}+\nu_{j(i)}+\theta_{k(ij)}+(\lambda_{1_{i}}+\lambda_{1_{j\left( i \right)}}+\lambda_{1_{k\left( ij \right)}})\times time +(\lambda_{2_{i}}+\lambda_{2_{j\left( i \right)}}+\lambda_{2_{k\left( ij \right)}})\times time^{2}+\epsilon$$

$time$ is the centered variable of year. $y_{ijkt}$is the response variable within the kth level of factor age within the jth level of factor sex within the ith level of factor province, at time t. $\alpha, \beta_{1}, \beta_{2}, \beta_{3} and \beta_{4}$ are the fixed effects for intercept, years of schooling, wealth index, urbanization and temperature ,respectively. $\omega_{i}, \nu_{j\left( i \right)}, and \theta_{k\left( ij \right)}$ are the random effects for ith level of province, jth level of sex nested within the ith level province, kth level of age group nested within the jth level of sex nested within the ith level province, respectively. Under the nested circumstances, as in the previous cases, $\lambda_{1_{i}}, \lambda_{1_{j\left( i \right)}}, \lambda_{1_{k\left( ij \right)}}, \lambda_{2_{i}}, \lambda_{2_{j\left( i \right)}} and \lambda_{2_{k\left( ij \right)}}$ are random effects for two quadratic form parameters. $\epsilon$ is an error term. Fixed effect coefficients have flat prior over the reals. Standard deviations of group-level random effects and error term have half student-t prior with 3 degrees of freedom and a scale parameter that depends on the standard deviation of the response.

In the second step, which is complementary to the first step, the age-spatial-temporal model ([5](#_ENREF_5)) consider the temporal, spatial, and age dependencies using the residuals of the first model that cannot capture this information. This is done using a local regression in three dimensions on residuals. The calculation process of this step is based on the posterior samples in Bayesian model. Finally estimate the mean salt intake and its 95% uncertainty interval from the 2·5th and 97·5th percentiles based on results of second model, across sex, age, year, and province.

***Age Standardization of results***

In order to remove the age effect, all estimates were age standardized using the population of Iran tabulated by sex, age at national level in 2016.

***Model Validity***

For assessing the validity of the model for estimation of mean salt intake, 10% of data points randomly selected and omitted; then, the model repeated for the remaining 90% of data. This process repeated five times. Afterwards, the overlap between confidence interval of the deleted data and uncertainty interval of estimates of the model repeated for the remaining 90% of data was evaluated, and the average of 86·5% overlap was seen. Furthermore, the Root Mean Square Error (RMSE) was calculated for total data and 90% of data. The value of RMSE for total data was 0·608 and for 90% of the data was 0·630.

***References***

1. [Tanaka](https://pubmed.ncbi.nlm.nih.gov/?term=Tanaka+T&cauthor_id=11850766) [T](https://pubmed.ncbi.nlm.nih.gov/11850766/#affiliation-1) ,  [Okamura](https://pubmed.ncbi.nlm.nih.gov/?term=Okamura+T&cauthor_id=11850766) T, [Miura](https://pubmed.ncbi.nlm.nih.gov/?term=Miura+K&cauthor_id=11850766) K, [Kadowaki](https://pubmed.ncbi.nlm.nih.gov/?term=Kadowaki+T&cauthor_id=11850766) T, [Ueshima](https://pubmed.ncbi.nlm.nih.gov/?term=Ueshima+H&cauthor_id=11850766) H, [Nakagawa](https://pubmed.ncbi.nlm.nih.gov/?term=Nakagawa+H&cauthor_id=11850766) H, et al. A simple method to estimate populational 24-h urinary sodium and potassium excretion using a casual urine specimen. J Hum Hypertens. 2002; 16(2): 97-103.

2. Ji C, Sykes L, Paul C, Dary O, Legetic B, Campbell NR, et al. Systematic review of studies comparing 24-hour and spot urine collections for estimating population salt intake. Revista panamericana de salud publica = Pan American journal of public health. 2012; 32(4):307-15.

3. Imhoff-Kunsch B, Flores R, Dary O, Martorell R. Methods of using household consumption and expenditures survey (HCES) data to estimate the potential nutritional impact of fortified staple foods. Food and nutrition bulletin. 2012;33(3 Suppl):S185-9.

4. Land M-A, Webster J, Christoforou A, Praveen D, Jeffery P, Chalmers J, et al. Salt intake assessed by 24 h urinary sodium excretion in a random and opportunistic sample in Australia. BMJ open. 2014;4(1):e003720-e.

5. Foreman KJ, Lozano R, Lopez AD, Murray CJ. Modeling causes of death: an integrated approach using CODEm. Population health metrics. 2012;10:1.

6. Nazeri P, Mirmiran P, Delshad H, Hedayati M, Azizi F. Evaluation of Urinary Iodine Concentration and Iodine Content of Households Salt in South of Tehran. IJEM. 2010;12(3):294-9.

7. Ghadimi R, Taheri H, Suzuki S, Kashifard M, Hosono A, Esfandiary I, et al. Host and environmental factors for gastric cancer in Babol, the Caspian Sea Coast, Iran. European journal of cancer prevention : the official journal of the European Cancer Prevention Organisation (ECP). 2007;16(3):192-5.

8. Mirzaei M, Soltaniz M, Namayandeh M, GharahiGhehi N. Sodium and potassium intake of urban dwellers: nothing changed in Yazd, Iran. Journal of health, population, and nutrition. 2014;32(1):111-7.

9. Nazeri P, Mirmiran P, Mehrabi Y, Hedayati M, Delshad H, Azizi F. Evaluation of iodine nutritional status in Tehran, Iran: iodine deficiency within iodine sufficiency. Thyroid : official journal of the American Thyroid Association. 2010;20(12):1399-406.

10. Rafiei M, Boshtam M, Sarrafzadegan N, Seirafian S. The relation between salt intake and blood pressure among Iranians. Kuwait Medical Journal. 2008;40:191-5.

11. Nazeri P, Mirmiran P, Asghari G, Delshad H, Mehrabi Y, Hedayati M, et al. Differences between subjects with sufficient and deficient urinary iodine in an area of iodine sufficiency. Journal of endocrinological investigation. 2011;34(9):e302-7.

12. Motlagh Z, Mazloomy S, Mozaffari Khosravi H, Morowatisharifabad M, Askarshahi M. Salt Intake Among Women Refer to Medical Health Centers, Yazd, Iran. JSSU. 2011;19(4):550-60.

| **Supplementary** **Table-1:** Search Syntax |
| --- |
| **PubMed**  (("Sodium, Dietary"[Mesh] OR "Sodium Chloride"[Mesh] OR Salt[TIAB] OR Na[TIAB] OR sodium[TIAB]) AND ("Eating"[Mesh] OR "diet"[MeSH] OR diet*[TIAB] OR Intake*[TIAB] OR consum*[TIAB] OR ingest*[TIAB] OR eat*[TIAB] OR "urine"[Mesh] OR "urine"[Subheading] OR urin*[TIAB] OR excret*[TIAB])) AND (“Iran” [Mesh] OR Iran*[TIAB] OR Iran[Affiliation]) AND ((“2000/01/01” [PDAT]: “2016/12/31” [PDAT]) |
| **ISI Web of Knowledge**  TS= ((Sodium OR Salt OR Na) AND (Diet* OR Intake* OR consum* OR ingest* OR eat* OR urin* OR excret*)) AND (TS=(Iran*) OR AD=(Iran)). Time span=2000-2016 |
| **Scopus**  TITLE-ABS-KEY ((Sodium OR Salt OR Na) AND (Diet* OR Intake* OR consum* OR ingest* OR eat* OR urin* OR excret*)) AND (TITLE-ABS-KEY (Iran*) OR AFFIL (Iran)) AND PUBYEAR AFT 1999 AND PUBYEAR BEF 2017 |

| **Supplementary** **Table 2:** National and Sub-national surveys from 2000 and 2016 among Iranian population | | | | | | | | | | |
| --- | --- | --- | --- | --- | --- | --- | --- | --- | --- | --- |
| **Survey (abbreviation)/Title of published article (first author)** | **Year** | **Study province** | **Rural, Urban or Both** | **Sample size** | | **Age groups**  **(year)** | **Measure (Urine, Diet)** | **Urine method †** | **Diet method ‡** | **Mean±SD of salt intake** |
|  |  |  |  | Male | Female |  |  |  |  |  |
| Comprehensive Project on Household Food Pattern and Nutritional Status | 2000 | All province | Both | 4,699 | 8,585 | 25-34, 35-44, 45-54, 55-64, 65-74, 75-84 and ≥85 | Diet | - | 1 | 10.9±2.7 |
|  | 2001 | All province | Both | 143,486 | 249,188 | 25-34, 35-44, 45-54, 55-64, 65-74, 75-84 and ≥85 | Diet | - | 1 | 10.2±2.6 |
|  | 2002 | All province | Both | 83,871 | 132,446 | 25-34, 35-44, 45-54, 55-64, 65-74, 75-84 and ≥85 | Diet | - | 1 | 10.0±3.1 |
| Persian Gulf Healthy Heart Study (PGHHS) | 2012 | Bushehr | Urban | 408 | 458 | 55-64, 65-74, 75-84 and ≥85 | Diet | - | 3 | 6.3±1.0 |
| Yazd Healthy Heart Program (YHHP) | 2003 | Yazd | Urban | 27 | 28 | 25-34, 35-44, 45-54, 55-64, 65-74 | Diet | - | 3 | 10.1±2.1 |
|  | 2004 | Yazd | Urban | 40 | 19 | 25-34, 35-44, 45-54, 55-64, 65-74 | Diet | - | 3 | 11.5±2.5 |
| Isfahan salt study | 2001 | Isfahan | Urban | 45 | 80 | 25-34, 35-44, 45-54, 55-64, 65-74, 75-84 | Urine | 1 | - | 9.3±1.5 |
|  | 2007 | Isfahan | Urban | 181 | 250 | 25-34, 35-44, 45-54, 55-64, 65-74, 75-84 | Urine | 1 | - | 9.2±1.2 |
|  | 2013 | Isfahan | Urban | 335 | 386 | 25-34, 35-44, 45-54, 55-64, 65-74 | Urine | 1 | - | 9.7±1.7 |
| The Tehran Lipid and Glucose Study (TLGS) | 2005 | Tehran | Urban | 35 | 70 | 25-34, 35-44, 45-54, 55-64, 65-74, 75-84 | Diet | - | 2 | 10.3±0.9 |
|  | 2006 | Tehran | Urban | 42 | 14 | 25-34, 45-54, 55-64 | Diet | - | 2 | 10.7±1.3 |
|  | 2007 | Tehran | Urban | 167 | 187 | 25-34, 35-44, 45-54, 55-64, 65-74 | Diet | - | 2 | 10.4±0.9 |
|  | 2008 | Tehran | Urban | 590 | 702 | 25-34, 35-44, 45-54, 55-64, 65-74, 75-84 and ≥85 | Diet | - | 2 | 8.6±1.1 |
|  | 2009 | Tehran | Urban | 435 | 614 | 25-34, 35-44, 45-54, 55-64, 65-74, 75-84 and ≥85 | Diet | - | 2 | 9.2±0.4 |
|  | 2010 | Tehran | Urban | 311 | 364 | 25-34, 35-44, 45-54, 55-64, 65-74 | Diet | - | 2 | 8.7±1.3 |
|  | 2011 | Tehran | Urban | 482 | 577 | 25-34, 35-44, 45-54, 55-64, 65-74, 75-84 and ≥85 | Diet | - | 2 | 8.5±1.1 |
|  | 2012 | Tehran | Urban | 701 | 1,066 | 25-34, 35-44, 45-54, 55-64, 65-74, 75-84 and ≥85 | Diet | - | 2 | 8.8±0.6 |
|  | 2013 | Tehran | Urban | 664 | 741 | 25-34, 35-44, 45-54, 55-64, 65-74, 75-84 and ≥85 | Diet | - | 2 | 9.0±0.8 |
|  | 2014 | Tehran | Urban | 817 | 857 | 25-34, 35-44, 45-54, 55-64, 65-74, 75-84 and ≥85 | Diet | - | 2 | 8.6±0.9 |
|  | 2015 | Tehran | Urban | 11 | 8 | 25-34, 35-44, 45-54, 55-64 | Diet | - | 2 | 8.5±1.5 |
| Isfahan Healthy Heart Program (IHHP) | 2007 | Isfahan | Urban | 622 | 628 | 25-34, 35-44, 45-54, 55-64, 65-74, 75-84 and ≥85 | Diet | - | 3 | 8.7±3.0 |
| Urban Health Equity assessment and Response Tool (Urban Heart-phase1) | 2007 | Tehran | Urban | 2,167 | 2,384 | 25-34, 35-44, 45-54, 55-64, 65-74, 75-84 and ≥85 | Diet | - | 1 | 10.2±1.7 |
| Mashhad stroke and heart atherosclerotic disorder (MASHAD) | 2007 | Mashhad | Urban | 97 | 150 | 25-34, 35-44, 45-54, 55-64, 65-74 | Diet | - | 2 | 6.0±1.5 |
|  | 2008 | Mashhad | Urban | 2457 | 3695 | 25-34, 35-44, 45-54, 55-64, 65-74, and ≥85 | Diet | - | 2 | 5.5±0.97 |
| Non-Communicable Disease Surveillance Survey (NCDSS) | 2016 | All province | Both | 8,670 | 9,962 | 25-34, 35-44, 45-54, 55-64, 65-74, 75-84 and ≥85 | Urine | 2 | - | 9.52±2.4 |
| Household Income & Expenditure study (HIEs) | 2004 | All province | Both | 25,721 | 25,804 | 25-34, 35-44, 45-54, 55-64, 65-74, 75-84 and ≥85 | Diet | - | 4 | 7.5±2.6 |
|  | 2005 | All province | Both | 28,325 | 28,645 | 25-34, 35-44, 45-54, 55-64, 65-74, 75-84 and ≥85 | Diet | - | 4 | 8.3±2.6 |
|  | 2006 | All province | Both | 32,367 | 33,142 | 25-34, 35-44, 45-54, 55-64, 65-74, 75-84 and ≥85 | Diet | - | 4 | 7.8±2.3 |
|  | 2007 | All province | Both | 32,699 | 33,476 | 25-34, 35-44, 45-54, 55-64, 65-74, 75-84 and ≥85 | Diet | - | 4 | 8.0± 2.5 |
|  | 2008 | All province | Both | 40,480 | 41,480 | 25-34, 35-44, 45-54, 55-64, 65-74, 75-84 and ≥85 | Diet | - | 4 | 7.7±2.5 |
|  | 2009 | All province | Both | 38,478 | 40,018 | 25-34, 35-44, 45-54, 55-64, 65-74, 75-84 and ≥85 | Diet | - | 4 | 7.8±2.6 |
|  | 2010 | All province | Both | 40,316 | 42,254 | 25-34, 35-44, 45-54, 55-64, 65-74, 75-84 and ≥85 | Diet | - | 4 | 8.2±2.7 |
|  | 2011 | All province | Both | 40,943 | 43,332 | 25-34, 35-44, 45-54, 55-64, 65-74, 75-84 and ≥85 | Diet | - | 4 | 8.2±2.7 |
|  | 2012 | All province | Both | 40,792 | 43,644 | 25-34, 35-44, 45-54, 55-64, 65-74, 75-84 and ≥85 | Diet | - | 4 | 8.3± 2.7 |
|  | 2013 | All province | Both | 39,644 | 40,737 | 25-34, 35-44, 45-54, 55-64, 65-74, 75-84 and ≥85 | Diet | - | 4 | 8.5±2.8 |
|  | 2014 | All province | Both | 40,038 | 41,829 | 25-34, 35-44, 45-54, 55-64, 65-74, 75-84 and ≥85 | Diet | - | 4 | 8.3±2.8 |
|  | 2015 | All province | Both | 40,183 | 42,424 | 25-34, 35-44, 45-54, 55-64, 65-74, 75-84 and ≥85 | Diet | - | 4 | 8.0±2.7 |
| Evaluation of Urinary Iodine Concentration and Iodine Content of Households Salt in South of Tehran ([6](#_ENREF_6)) | 2009 | Tehran | Urban | 62 | 85 | 25-34, 35-44 | Urine | 1 | - | 10.4±5.3 |
| Host and environmental factors for gastric cancer in Babol, the Caspian Sea Coast, Iran ([7](#_ENREF_7)) | 2004 | Mazandaran | Urban | 50 | 80 | 25-34, 35-44, 45-54, 55-64, 65-74, 75-84 | Diet | - | 2 | 10.9±3.5 |
| Sodium and Potassium Intake of Urban Dwellers: Nothing Changed in Yazd, Iran ([8](#_ENREF_8)) | 2004 | Yazd | Urban | 138 | 75 | 25-34, 35-44, 45-54, 55-64, 65-74 | Urine | 1 | - | 9.4±4.3 |
| Evaluation of Iodine Nutritional Status in Tehran, Iran: Iodine Deficiency Within Iodine Sufficiency ([9](#_ENREF_9)) | 2009 | Tehran | Urban | 242 | 397 | 25-34, 35-44 | Urine | 1 | - | 7.6±3.2 |
| The Relation between Salt Intake and Blood Pressure among Iranians ([10](#_ENREF_10)) | 2001 | Isfahan | Urban | 304 | 608 | 25-34, 35-44, 45-54, 55-64 | Urine | 1 | - | 9.9±2.9 |
| Differences between subjects with sufficient and deficient urinary iodine in an area of iodine sufficiency ([11](#_ENREF_11)) | 2009 | Isfahan | Urban | 242 | 397 | 25-34, 35-44 | Urine | 1 | - | 7.7±3.2 |
| Salt Intake Among Women Refer to Medical Health Centers, Yazd, Iran, 2011 ([12](#_ENREF_12)) | 2010 | Yazd | Urban | - | 247 | 25-34, 35-44 | Urine | 3 | - | 10.1±3.0 |
| †1=24-h urine. 2=Completeness of collection validated by PABA administration. 3. Spot urine sample  ‡1=Multiple (≥2 days) short-term (up to 1 wk) diet recalls/records. 2=Food frequency questionnaire. 3=Single short-term diet record/recall. 4=Household Income and Expenditure Survey. | | | | | | | | | | |

**Supplementary figure 1:** Number of data points by province and year.

**Supplementary figure 2:** Trends in age-standardized mean salt intake in women and by province from 2000 to 2016 in Iran.

**Supplementary figure 3:** Trends in age-standardized mean salt intake in men and by province from 2000 to 2016 in Iran.

**Supplementary figure 4:** Ranking of age-standardized mean salt intake in women and by province in 2000 and 2016 in Iran.

**Supplementary figure 5:** Ranking of age-standardized mean salt intake in men and by province in 2000 and 2016 in Iran.

**Supplementary figure 6:** Age-standardized mean and 95% uncertainty interval salt intake by sex in 2000 and 2016 in Iran.
