# Supplementary figures and images for: National and sub-national trends of salt intake in Iranians from 2000 to 2016: a systematic analysis
Source: Arch Public Health. 2022 Apr 13;80:120. doi: 10.1186/s13690-022-00871-w (PMC9006553; doi:10.1186/s13690-022-00871-w)

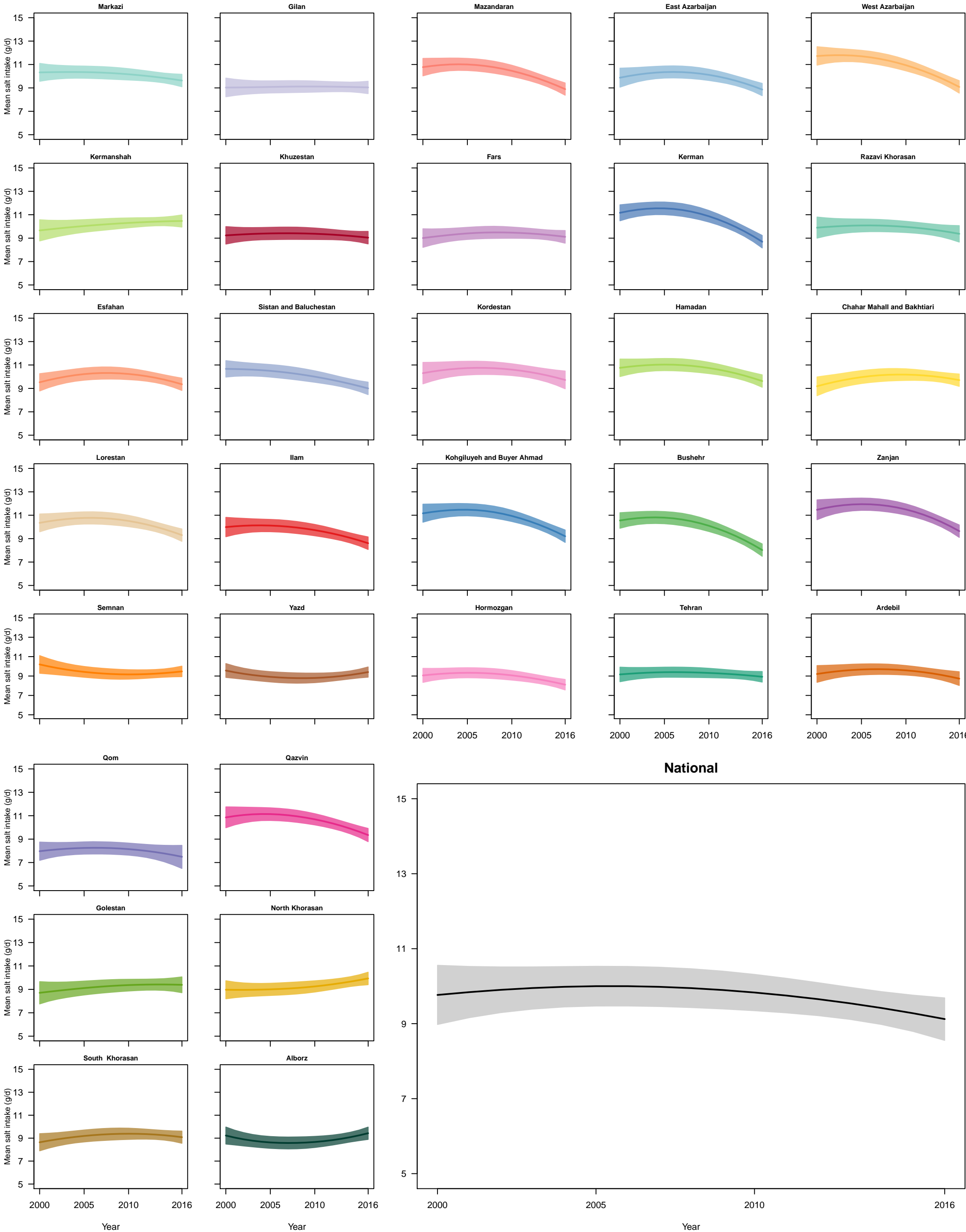

Supplement: Supplementary file 3 — Additional file 3. [file 13690_2022_871_MOESM3_ESM.pdf]

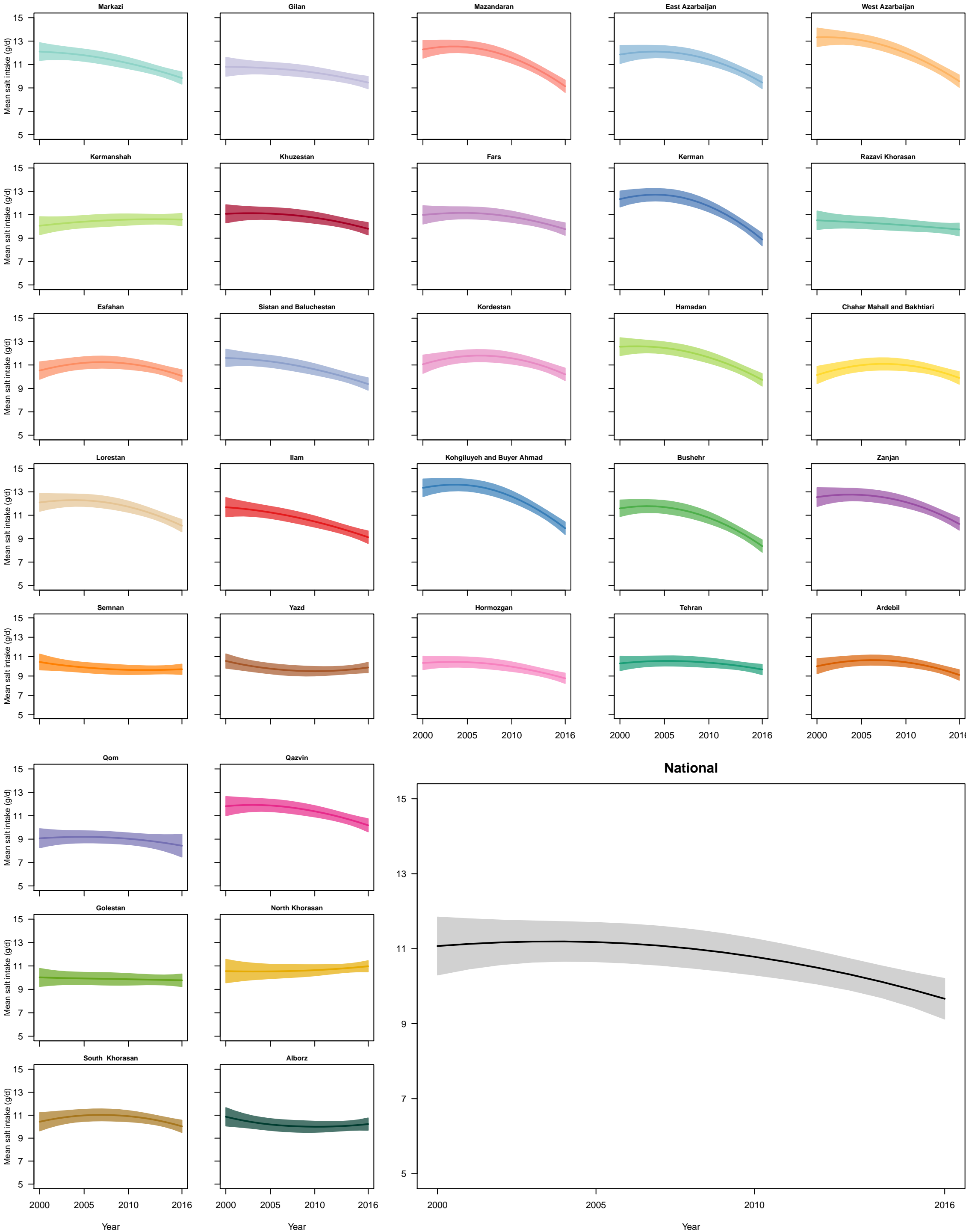

Supplement: Supplementary file 4 — Additional file 4. [file 13690_2022_871_MOESM4_ESM.pdf]

2000

Female

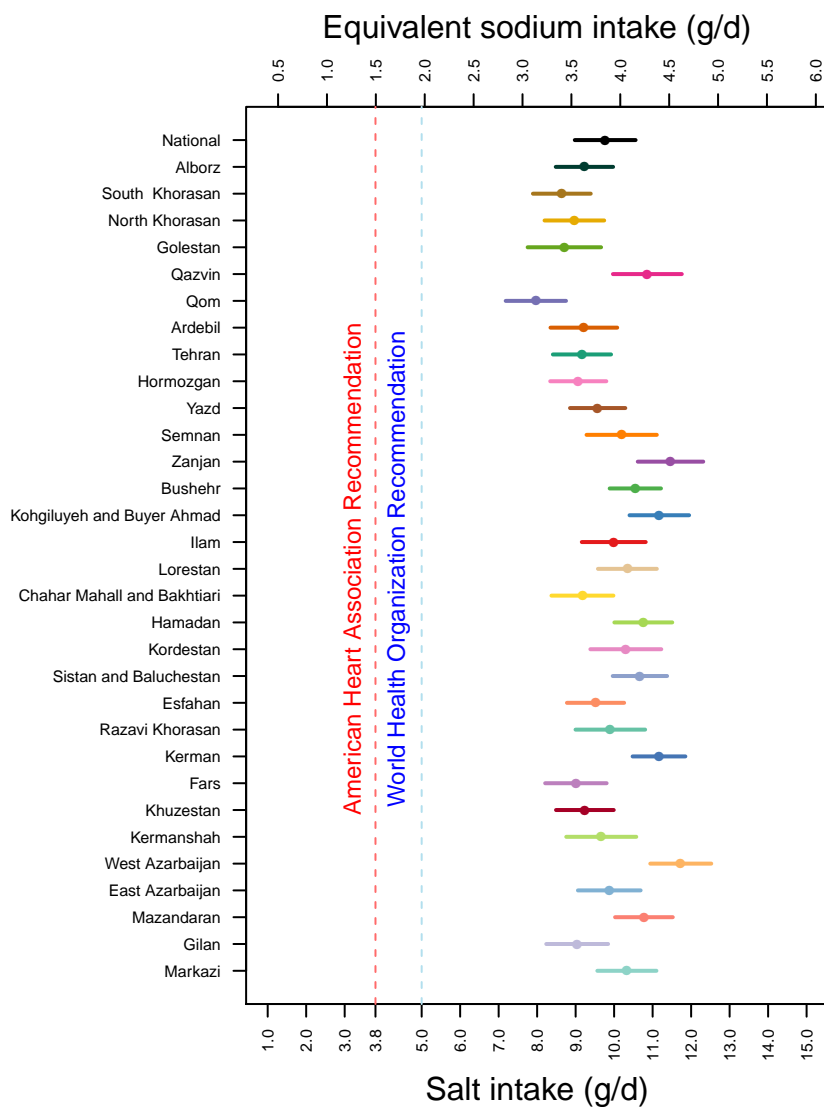

2016

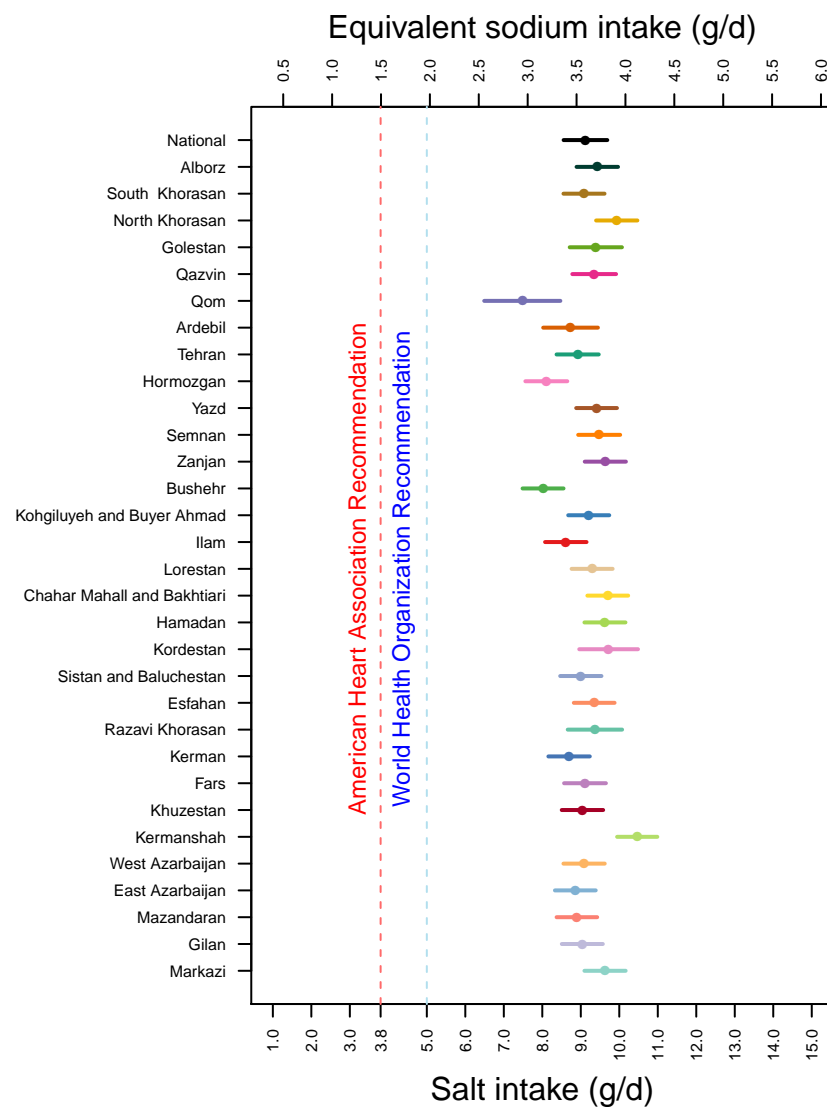

Male

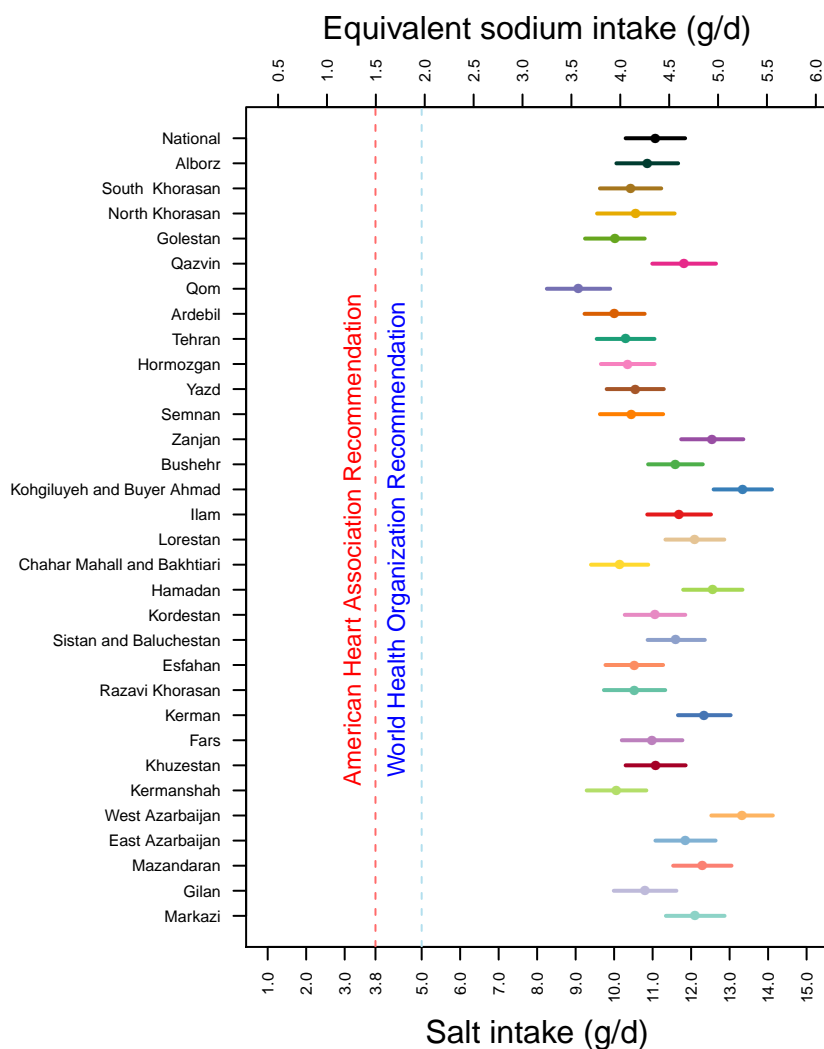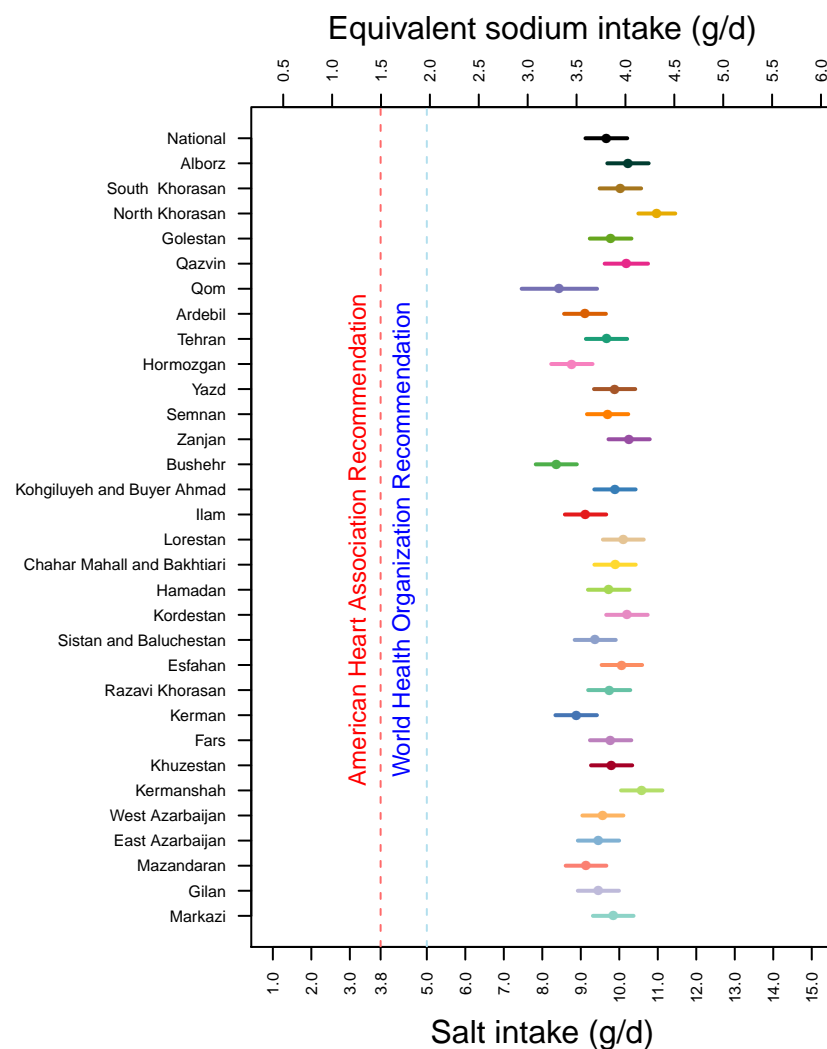

Supplement: Supplementary file 7 — Additional file 7. [file 13690_2022_871_MOESM7_ESM.pdf]
