# Supplementary material for: National and sub-national trends of salt intake in Iranians from 2000 to 2016: a systematic analysis
Source: Arch Public Health. 2022 Apr 13;80:120. doi: 10.1186/s13690-022-00871-w (PMC9006553; doi:10.1186/s13690-022-00871-w)
Supplement: Supplementary file 5 — Additional file 5. [file 13690_2022_871_MOESM5_ESM.pdf]

Female

2000

Mean salt intake (g/d) (95% UI)	Province
11.7 (10.9 to 12.5)	1. Azerbaijan, West
11.5 (10.6 to 12.3)	2. Zanjan
11.2 (10.5 to 11.9)	3. Kerman
11.2 (10.4 to 11.9)	4. Kohgiluyeh and Boyer−Ahmad
10.9 (10.0 to 11.8)	5. Qazvin
10.8 (10.0 to 11.5)	6. Mazandaran
10.8 (10.0 to 11.5)	7. Hamadan
10.7 (10.0 to 11.4)	8. Sistan and Baluchistan
10.5 (9.9 to 11.2)	9. Bushehr
10.3 (9.6 to 11.1)	10. Lorestan
10.3 (9.6 to 11.1)	11. Markazi
10.3 (9.4 to 11.2)	12. Kordestan
10.2 (9.3 to 11.1)	13. Semnan
10.0 (9.2 to 10.8)	14. Ilam
9.9 (9.0 to 10.8)	15. Khorasan, Razavi
9.9 (9.1 to 10.7)	16. Azerbaijan, East
9.7 (8.8 to 10.6)	17. Kermanshah
9.6 (8.9 to 10.3)	18. Yazd
9.5 (8.8 to 10.3)	19. Isfahan
9.2 (8.5 to 10.0)	20. Khuzestan
9.2 (8.5 to 10.0)	21. Alborz
9.2 (8.3 to 10.1)	22. Ardabil
9.2 (8.4 to 10.0)	23. Chahar Mahaal and Bakhtiari
9.2 (8.4 to 9.9)	24. Tehran
9.1 (8.3 to 9.8)	25. Hormozgan
9.0 (8.2 to 9.8)	26. Gilan
9.0 (8.2 to 9.8)	27. Fars
9.0 (8.2 to 9.7)	28. Khorasan, North
8.7 (7.8 to 9.7)	29. Golestan
8.6 (7.9 to 9.4)	30. Khorasan, South
8.0 (7.2 to 8.8)	31. Qom

2016

Province	Mean salt intake (g/d) (95% UI)	% Change (95% UI)
1. Kermanshah	10.5 (9.9 to 11.0)	8.4% (−6.0 to 25.5)
2. Khorasan, North	9.9 (9.4 to 10.5)	10.8% (−3.5 to 27.8)
3. Kordestan	9.7 (9.0 to 10.5)	−5.7% (−20.2 to 11.8)
4. Chahar Mahaal and Bakhtiari	9.7 (9.2 to 10.2)	5.7% (−8.1 to 22.2)
5. Zanjan	9.6 (9.1 to 10.2)	−15.9% (−26.1 to −4.2)
6. Markazi	9.6 (9.1 to 10.2)	−6.7% (−18.1 to 6.3)
7. Hamadan	9.6 (9.1 to 10.2)	−10.5% (−21.0 to 1.5)
8. Semnan	9.5 (8.9 to 10.0)	−7.1% (−19.6 to 8.0)
9. Alborz	9.4 (8.9 to 10.0)	2.2% (−10.9 to 17.5)
10. Yazd	9.4 (8.9 to 9.9)	−1.6% (−13.7 to 12.3)
11. Golestan	9.4 (8.7 to 10.1)	7.8% (−9.8 to 29.9)
12. Khorasan, Razavi	9.4 (8.7 to 10.1)	−5.3% (−19.9 to 12.0)
13. Isfahan	9.4 (8.8 to 9.9)	−1.8% (−14.1 to 12.6)
14. Qazvin	9.3 (8.8 to 9.9)	−13.9% (−25.3 to −0.5)
15. Lorestan	9.3 (8.8 to 9.8)	−10.1% (−21.1 to 2.6)
16. Kohgiluyeh and Boyer−Ahmad	9.2 (8.7 to 9.7)	−17.6% (−27.4 to −6.3)
17. Fars	9.1 (8.6 to 9.7)	1.1% (−12.6 to 17.6)
18. Azerbaijan, West	9.1 (8.5 to 9.6)	−22.5% (−31.7 to −12.0)
19. Khorasan, South	9.1 (8.6 to 9.6)	5.1% (−9.0 to 21.9)
20. Khuzestan	9.0 (8.5 to 9.6)	−2.1% (−14.9 to 12.8)
21. Gilan	9.0 (8.5 to 9.6)	0.1% (−13.6 to 16.2)
22. Sistan and Baluchistan	9.0 (8.5 to 9.5)	−15.7% (−25.6 to −4.3)
23. Tehran	8.9 (8.4 to 9.5)	−2.6% (−15.6 to 12.6)
24. Mazandaran	8.9 (8.4 to 9.4)	−17.5% (−27.4 to −6.0)
25. Azerbaijan, East	8.9 (8.3 to 9.4)	−10.3% (−22.1 to 3.6)
26. Ardabil	8.7 (8.0 to 9.4)	−5.2% (−20.4 to 13.2)
27. Kerman	8.7 (8.2 to 9.2)	−22.1% (−31.2 to −11.9)
28. Ilam	8.6 (8.1 to 9.1)	−13.8% (−25.4 to −0.1)
29. Hormozgan	8.1 (7.6 to 8.6)	−10.5% (−22.8 to 3.7)
30. Bushehr	8.0 (7.5 to 8.6)	−23.9% (−33.3 to −13.5)
31. Qom	7.5 (6.5 to 8.5)	−6.0% (−25.8 to 17.9)

Ascending
Monotone
Descending
