# Supplementary material for: National and sub-national trends of salt intake in Iranians from 2000 to 2016: a systematic analysis
Source: Arch Public Health. 2022 Apr 13;80:120. doi: 10.1186/s13690-022-00871-w (PMC9006553; doi:10.1186/s13690-022-00871-w)
Supplement: Supplementary file 6 — Additional file 6. [file 13690_2022_871_MOESM6_ESM.pdf]

Male

2000

Mean salt intake (g/d) (95% UI)	Province
13.3 (12.6 to 14.1)	1. Kohgiluyeh and Boyer−Ahmad
13.3 (12.5 to 14.1)	2. Azerbaijan, West
12.6 (11.8 to 13.3)	3. Hamadan
12.5 (11.7 to 13.4)	4. Zanjan
12.3 (11.7 to 13.0)	5. Kerman
12.3 (11.5 to 13.0)	6. Mazandaran
12.1 (11.3 to 12.9)	7. Markazi
12.1 (11.3 to 12.9)	8. Lorestan
11.9 (11.1 to 12.6)	9. Azerbaijan, East
11.8 (11.0 to 12.6)	10. Qazvin
11.7 (10.9 to 12.5)	11. Ilam
11.6 (10.9 to 12.4)	12. Sistan and Baluchistan
11.6 (10.9 to 12.3)	13. Bushehr
11.1 (10.3 to 11.9)	14. Khuzestan
11.1 (10.3 to 11.8)	15. Kordestan
11.0 (10.2 to 11.8)	16. Fars
10.9 (10.1 to 11.7)	17. Alborz
10.8 (10.0 to 11.6)	18. Gilan
10.6 (9.6 to 11.6)	19. Khorasan, North
10.6 (9.8 to 11.3)	20. Yazd
10.5 (9.7 to 11.3)	21. Khorasan, Razavi
10.5 (9.8 to 11.3)	22. Isfahan
10.5 (9.6 to 11.3)	23. Semnan
10.4 (9.6 to 11.2)	24. Khorasan, South
10.3 (9.7 to 11.1)	25. Hormozgan
10.3 (9.5 to 11.0)	26. Tehran
10.1 (9.4 to 10.9)	27. Chahar Mahaal and Bakhtiari
10.1 (9.3 to 10.8)	28. Kermanshah
10.0 (9.2 to 10.8)	29. Golestan
10.0 (9.2 to 10.8)	30. Ardabil
9.1 (8.3 to 9.9)	31. Qom

2016

Province	Mean salt intake (g/d) (95% UI)	% Change (95% UI)
1. Khorasan, North	11.0 (10.5 to 11.5)	3.9% (−9.3 to 19.9)
2. Kermanshah	10.6 (10.0 to 11.1)	5.2% (−7.3 to 19.7)
3. Zanjan	10.3 (9.7 to 10.8)	−18.3% (−27.2 to −8.1)
4. Alborz	10.2 (9.7 to 10.8)	−5.9% (−16.9 to 7.0)
5. Kordestan	10.2 (9.7 to 10.7)	−7.8% (−18.5 to 4.5)
6. Qazvin	10.2 (9.6 to 10.7)	−13.8% (−23.9 to −2.2)
7. Lorestan	10.1 (9.6 to 10.6)	−16.5% (−25.6 to −6.1)
8. Isfahan	10.1 (9.5 to 10.6)	−4.4% (−15.4 to 8.4)
9. Khorasan, South	10.0 (9.5 to 10.6)	−3.8% (−15.5 to 9.7)
10. Chahar Mahaal and Bakhtiari	9.9 (9.4 to 10.4)	−2.4% (−14.1 to 10.9)
11. Kohgiluyeh and Boyer−Ahmad	9.9 (9.4 to 10.4)	−25.9% (−33.7 to −17.2)
12. Yazd	9.9 (9.3 to 10.4)	−6.4% (−17.3 to 6.2)
13. Markazi	9.8 (9.3 to 10.4)	−18.6% (−27.6 to −8.6)
14. Khuzestan	9.8 (9.3 to 10.3)	−11.6% (−21.9 to 0.4)
15. Golestan	9.8 (9.2 to 10.3)	−2.5% (−14.5 to 11.6)
16. Fars	9.8 (9.2 to 10.3)	−11.1% (−21.5 to 1.1)
17. Khorasan, Razavi	9.7 (9.2 to 10.3)	−7.5% (−18.9 to 5.7)
18. Hamadan	9.7 (9.2 to 10.3)	−22.5% (−31.1 to −13.0)
19. Semnan	9.7 (9.2 to 10.2)	−7.2% (−18.7 to 6.3)
20. Tehran	9.7 (9.1 to 10.2)	−6.1% (−17.3 to 6.9)
21. Azerbaijan, West	9.6 (9.0 to 10.1)	−28.2% (−36.0 to −19.3)
22. Azerbaijan, East	9.5 (8.9 to 10.0)	−20.2% (−29.4 to −9.7)
23. Gilan	9.5 (8.9 to 10.0)	−12.5% (−23.2 to 0.0)
24. Sistan and Baluchistan	9.4 (8.8 to 9.9)	−19.2% (−28.5 to −8.8)
25. Mazandaran	9.1 (8.6 to 9.7)	−25.6% (−34.0 to −16.2)
26. Ilam	9.1 (8.6 to 9.7)	−21.9% (−31.4 to −11.1)
27. Ardabil	9.1 (8.6 to 9.7)	−9.0% (−20.6 to 4.6)
28. Kerman	8.9 (8.3 to 9.4)	−28.0% (−36.0 to −19.2)
29. Hormozgan	8.8 (8.2 to 9.3)	−15.4% (−25.5 to −3.6)
30. Qom	8.4 (7.5 to 9.4)	−7.0% (−24.6 to 14.2)
31. Bushehr	8.4 (7.8 to 8.9)	−27.8% (−36.4 to −18.3)

Ascending
Monotone
Descending
