# Supplementary material for: National and sub-national trends of salt intake in Iranians from 2000 to 2016: a systematic analysis
Source: Arch Public Health. 2022 Apr 13;80:120. doi: 10.1186/s13690-022-00871-w (PMC9006553; doi:10.1186/s13690-022-00871-w)
Supplement: Supplementary file 2 — Additional file 2. [file 13690_2022_871_MOESM2_ESM.pdf]

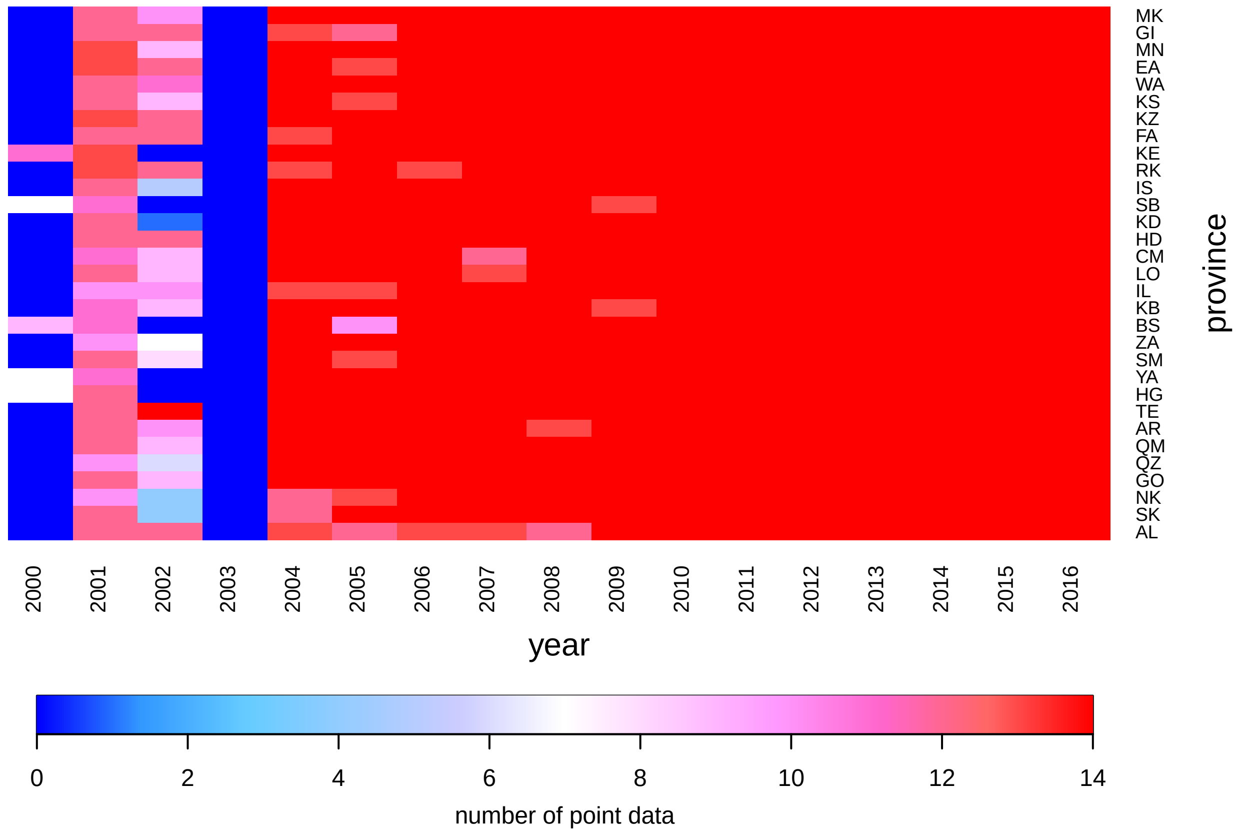
MK
GI
MN
EA
WA
KS
KZ
FA
KE
RK
IS
SB
KD
HD
CM
LO
IL
KB
BS
ZA
SM
YA
HG
TE
AR
QM
QZ
GO
NK
SK
AL
province
2000
2001
2002
2003
2004
2005
2006
2007
2008
2009
2010
2011
2012
2013
2014
2015
2016
year
0
2
4
6
8
10
12
14
number of point data
